# Involving YOUNG people with Type 1 Diabetes in reSearch and the development of healTh cARe activities

**DOI:** 10.1093/eurpub/ckac131.138

**Published:** 2022-10-25

**Authors:** K Ganahl, K Paldán, A Bernhard

**Affiliations:** 1 Research Division, Agency of Preventive and Social Medicine, Bregenz, Austria; 2 Research Centre for User Centred Technologies, Vorarlberg University of Applied Science, Dornbirn, Austria; 3 Research & Development, aha Youth Info, Dornbirn, Austria

## Abstract

**Background:**

Typ1 Diabetes (T1D) us a complicated condition that requires constant monitoring and making many decisions. Living with T1D means to check blood sugar levels, inject oneself insulin, and be careful what you eat. In the life of a young adult there are a lot of things going on and youngsters don't want to control themselves all day and night. They want to rebel and feel free.

**Methods:**

Teenagers living with T1D are actively involved in the research process from data collection to data interpretation. Following the photo voice method the teenagers documented their everyday life with T1D by photographs and recordings (Summer 2021), and discussed their need and resources in group discussions. The visual and audio-data was structured on a muti-touchscreen for which a software was developed during the project. From the inductively formed categories, measures were derived together with the young people and transferred into an action plan, which was presented and discussed with decision-makers.

**Results:**

During the photo voice process, the teenagers identified 26 categories (i.e. nutrition, coping strategies, autonomy, etc.) that were important for describing their life with diabetes and there categories were categorized into 4 dimension: psycho-social, time, subject and space. The project-team used a systematic action planning process to develop 9 goals for an action plan to improve their life with T1D. They presented their action plan to local political leaders and stakeholders from different setting, such as health care and youth work. The responses during the stakeholder dialog were positively and there was agreement to achieve the defined goals.

**Conclusions:**

The photo-voice method was helpful in immersing the participants in the lives of young people with type 1 diabetes. Structuring and organizing the visual- and audio-data together was beneficial for the participatory process. Together, an action plan could be developed and discussed with decision makers.

**Key messages:**

• The photo voive method using digital tools (like smartphones and multitouchscreen) is a route to empower young people and give them a voice.

• The participating patients are experts for their bodies and the disease and should be perceived and taken seriously as such.

